# Dynamics of Absences Due to Respiratory Infections, Including COVID-19, Among Medical Staff in a Regional Pediatric Hospital

**DOI:** 10.3390/healthcare13050563

**Published:** 2025-03-05

**Authors:** Maria Valentina Popa, Irina Luciana Gurzu, Dana Elena Mîndru, Bogdan Gurzu, Claudia Mariana Handra, Elkan Eva-Maria, Iulia Olaru, Dana Teodora Anton-Păduraru, Cezarina Warter, Letiția Doina Duceac

**Affiliations:** 1 Doctoral School of Biomedical Sciences, “Dunărea de Jos” University of Galați, Galați, 47 Domnească Street, 800008 Galați, Romania; maria_valentina_popa@yahoo.com; 2 Department of Preventive Medicine and Interdisciplinarity, Discipline of Occupational Health, “Gr. T. Popa” University of Medicine and Pharmacy, 700115 Iasi, Romania; 3 Department of Pediatrics, Faculty of Medicine, “Gr. T. Popa” University of Medicine and Pharmacy, 700115 Iasi, Romania; mindru.dana@umfiasi.ro (D.E.M.); dana.anton@umfiasi.ro (D.T.A.-P.); 4 Department of Morfofunctional Sciences, Faculty of Medicine, “Gr. T. Popa” University of Medicine and Pharmacy, 700115 Iasi, Romania; bgurzu@yahoo.com; 5 Occupational Medicine Department, “Carol Davila” University of Medicine and Pharmacy, 050474 Bucharest, Romania; claudia.handra@gmail.com; 6 Faculty of Medicine and Pharmacy, “Dunărea de Jos” University of Galați, 47 Domnească Street, 800008 Galați, Romania; cojocarumariaeva@yahoo.com (E.E.-M.); iulia_dabija@yahoo.com (I.O.); letimedr@yahoo.com (L.D.D.); 7 “Sf. Maria” Emergency Children’s Hospital, 700309 Iasi, Romania; c.dragomirescu@yahoo.com

**Keywords:** sick leave, absenteeism, respiratory infections, healthcare workers, COVID-19, management

## Abstract

**Background**: Respiratory infections pose a significant public health challenge, particularly among healthcare workers (HCWs). The COVID-19 pandemic exacerbated absenteeism due to respiratory illnesses, affecting healthcare workforce stability. Identifying factors influencing absenteeism is crucial for workforce resilience and effective care. **Methods**: This retrospective longitudinal study analyzed HCW absenteeism due to respiratory diseases from 2017 to 2023 at the “Sf. Maria” Children’s Emergency Hospital in Iași, Romania. Data from 3827 HCWs were examined, including demographic and occupational variables (age, gender, job role) and disease types. Statistical analyses (chi-square tests, ANOVA, and regression models) were conducted using SPSS to assess absenteeism trends and associated risk factors. **Results**: Sick leave peaked in 2020 (8322 days) and remained high in 2021 (8134 days), gradually decreasing in 2022–2023 but not returning to pre-pandemic levels (~5000 days/year). Nurses accounted for most leave days, while male staff and HCWs aged 41–50 were most affected. Seasonal variations showed higher absenteeism in transitional months and lower rates in summer. COVID-19 was the leading cause of absenteeism during the pandemic, with quarantine measures further increasing sick leave duration. **Conclusions**: Pediatric hospitals must strengthen infection control measures to protect HCWs and sustain care continuity. Preventive actions such as immunization, staff training, and health monitoring are critical in reducing absenteeism, maintaining a resilient workforce, and ensuring quality care during health crises.

## 1. Introduction

Due to frequent exposure to infected patients, healthcare workers (HCWs) face a high risk of respiratory illnesses [1,2,3], which range in severity [4,5]. Occupational factors further exacerbate these risks, impacting both staff well-being and continuity of care [6,7]. Absenteeism among HCWs due to respiratory illnesses [8] significantly affects efficiency and continuity of care [9], particularly during seasonal peaks and the COVID-19 pandemic [10].

The close patient contact required in pediatric practice increases HCWs’ risk of respiratory infections, leading to higher absenteeism [11,12] and disrupted care continuity [13,14]. As a result, frequent pathogen exposure places additional strain on pediatric healthcare teams by increasing sick leave rates [15,16,17,18]. This phenomenon is particularly pronounced during periods of elevated incidence of respiratory infections, both seasonal (such as influenza and common respiratory viral infections) [19,20,21] and emerging, such as COVID-19 [2].

In Romania, systemic healthcare challenges—including limited funding [22], workforce shortages [22,23], and inequitable access [24]—exacerbate absenteeism issues, particularly in pediatric hospitals. In addition, high infant mortality rates [23] and inefficient reforms influenced by political instability further contribute to the situation [25].

The COVID-19 pandemic highlighted the vulnerability of HCWs to respiratory infections [26], which had a significant effect on the continuity of healthcare services [27,28,29]. During the COVID-19 pandemic, the stress of parents of pediatric patients increased due to the restrictions and impact on health services, requiring clear communication about safety measures as well as support for families in coping with the situation [30,31]. Even in the post-pandemic period, the effects persist [32], highlighting the need for sustainable strategies to reduce absenteeism and protect the well-being of hospital staff [33].

Absenteeism among pediatric HCWs due to respiratory infections significantly impacts care quality and continuity. This study examines the dynamics of HCW absenteeism due to respiratory infections, including COVID-19, in a regional pediatric hospital in Romania. It aims to identify key trends and causes of sick leave.

The study fills an important gap in the literature by providing a detailed analysis of sickness absence due to respiratory infections in pediatric hospitals in a country with specific health system contexts, such as Romania, highlighting long-term trends and suggesting practical measures to reduce the impact on the health system.

The objective of this study is to analyze the phenomenon of absenteeism caused by respiratory infections, including those caused by the virus known as SARS-CoV-2, among medical staff in the largest pediatric hospital in the northeastern region of Romania, over a period of seven years (2017–2023). The study intends to assess sick leave trends, identify demographic and occupational factors, determine the typology of respiratory infections, and analyze the seasonality of absenteeism. The primary hypothesis of the study is that the absenteeism of pediatric HCWs due to respiratory infections increased significantly during the pandemic period (2020–2021) compared to the pre-pandemic period (2017–2019) and remained above the baseline level during the post-pandemic period (2022–2023) due to respiratory infectious diseases. The results obtained may contribute to the formulation of sustainable measures to reduce absenteeism and maintain continuity of pediatric care, thereby ensuring better healthcare system functioning during critical periods.

## 2. Materials and Methods

This retrospective longitudinal study was conducted over seven years, from 2017 to 2023, aiming to evaluate absenteeism caused by respiratory diseases (upper and lower respiratory tract infections and COVID-19) among healthcare personnel at the “Sf. Maria” Children’s Emergency Clinical Hospital in Iași. This hospital is the largest pediatric referral center in the northeastern region of Romania, serving a population of over 3 million people, with approximately one-fifth being children (https://insse.ro, accessed on 18 February 2025). The hospital provides highly specialized pediatric healthcare, including 13 medical departments (covering 10 specialties), 4 surgical departments (covering 5 specialties), an integrated outpatient clinic, a laboratory, a radiology unit, and an emergency unit. The hospital also has the status of a university-affiliated teaching hospital for medical students and resident doctors.

The study compares trends before, during, and after the pandemic, identifies demographic and occupational factors influencing absenteeism, and determines the typology of infections and their seasonality. The results provide useful insights to propose effective management strategies in occupational health to reduce the impact of absenteeism on the continuity and quality of medical care.

The study population comprised all hospital employees, including doctors, nurses, and auxiliary staff, out of a total of 3827 HCWs who had sick leave days for respiratory infections during 2017–2023. Inclusion criteria: medical leaves granted for respiratory diseases (upper and lower respiratory tract infections and COVID-19) between 2017 and 2023. Exclusion criteria: medical leaves granted for causes other than respiratory diseases, incomplete leave records, or unclear or duplicate disease codes. Data were obtained from occupational medicine registers (contained certified medical leave records for all hospital employees) and electronic medical leave recording systems (including detailed ICD-coded diagnoses for respiratory infections).

The analyzed variables included the following: (i) disease codes according to the International Classification of Diseases (ICD), to quickly find upper and lower respiratory tract infections and COVID-19; (ii) date and duration of medical leaves; (iii) demographic characteristics of the personnel (age, gender); and (iv) occupation (doctors, nurses, auxiliary staff).

The collected data were processed to eliminate errors, duplicates, and incomplete information. Disease codes were verified to ensure the exclusive inclusion of medical leaves caused by respiratory diseases and COVID-19. Leaves that did not meet the inclusion criteria were excluded. The dataset did not include self-reported absenteeism (i.e., HCWs who worked while symptomatic but did not take formal sick leave), which is acknowledged as a potential limitation.

Data analysis was conducted using SPSS statistical software (version 25.0). Descriptive statistics, including mean, standard deviation, and proportions, were used to summarize sick leave duration, occupational distribution, and seasonal trends. Comparative analyses were performed using chi-square tests to examine associations between categorical variables, such as occupation and absenteeism. Additionally, *t*-tests and ANOVA were applied to compare absenteeism rates across different years and job roles.

To evaluate long-term patterns, a time-series analysis was conducted to assess absenteeism trends over the study period. Absenteeism rates were also compared across three distinct timeframes: the pre-pandemic period (2017–2019), the pandemic period (2020–2021), and the post-pandemic period (2022–2023). Furthermore, a multivariate regression analysis was performed to adjust for potential confounders, including age, gender, occupation, and seasonal variations, ensuring a more accurate assessment of factors influencing absenteeism.

All statistical tests were two-tailed, and results were considered statistically significant at a *p*-value of less than 0.05.

The study complied with ethical standards in medical research. Personal data were anonymized. This study was realized in accordance with the Declaration of Helsinki. The use of the data was approved by the Ethics Committee of the Emergency Hospital for Children “Sf. Maria” Iasi (35983/13.12.2022).

## 3. Results

**Figure 1:**
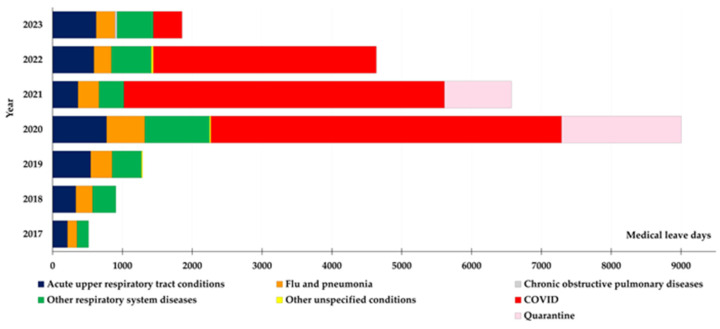
Number and distribution of sick leave days for respiratory infections per year.

Figure 1 shows the number and distribution of days of sick leave granted for respiratory infections based on medical certifications during the period 2017–2023. It is observed that 2020 recorded the highest number of days, exceeding 9000 days. After 2020, the number of days gradually decreased but remained considerably higher in 2021 and 2022. Even if absenteeism decreased in 2023 (below 2000), it remained higher than in 2017–2019. Also, from the graph in Figure 1, we can see that the proportions of different types of respiratory infections have changed over the years. Before the pandemic (2017–2019), most cases were acute upper respiratory tract infections (dark blue) and other chronic respiratory diseases (green). However, in 2020–2021, there was a sharp rise in COVID-19 cases (red), which made up most infections during this period, along with quarantine (pink) measures reflecting the strict regulations in place. Influenza and pneumonia (orange) became less common, likely due to preventive measures like mask-wearing and social distancing. By 2022–2023, COVID-19 cases had decreased, and other infections, such as acute and chronic respiratory conditions, became more noticeable.

Table 1 shows the averages of sick leave obtained for each of the years 2017–2023. Statistical analysis of the average number of sick leave days due to respiratory infections confirms the trends observed in Figure 1. In the pre-pandemic period (2017–2019), the average number of sick leave days was relatively stable, ranging from 4769 days (2017) to 5361 days (2018). In contrast, absenteeism reached significantly higher values during the COVID-19 pandemic (2020–2021), with 8322 days in 2020 and 8134 days in 2021. These values confirm the strong impact of the pandemic on HCWs, correlated with the increase in COVID-19 cases and quarantine measures. After the pandemic itself, in the post-pandemic period (2022–2023), a gradual decrease in absenteeism is observed, but it remains above pre-pandemic levels. In 2022, the average number of sick days was 5028, and in 2023, it dropped to 4332, suggesting a gradual return to normal but not to the initial 2017–2019 values.

To assess potential differences among the annual data, we applied a univariate one-way analysis of variance (ANOVA) using “Year” as the main factor. This approach provides a single, global test that evaluates whether the mean response differs across all years, inherently incorporating all data points simultaneously. Statistical analysis of the influence of the year variable on absenteeism revealed a significant effect (F(6, 3764) = 120.76, *p* < 0.001, η^2^ = 0.161) (Table 2). The Partial Eta Squared suggests that the year explains about 16.1% of the variance in sick leave days. The *p*-value indicates a statistically significant effect of the year on absenteeism (sick leave days). This means that at least one year’s mean sick leave days significantly differ from the others.

Given the results of the univariate ANOVA with “Year” as the main factor, we considered it helpful to perform post hoc pairwise comparisons using the LSD (Least Significant Difference) method. This combination (univariate ANOVA plus LSD) suits our primary interest, simple pairwise comparisons (e.g., Year 1 vs. Year 2), and our study design involves independent measurements across years rather than repeated measures on the same subjects. In order to provide a visualization of the significant differences between the pandemic period and the other time intervals, we also present the comparative analysis of the average number of days of sick leave for respiratory infections between 2017 and 2023 (Table 3).

The year 2020, which marks the beginning of the COVID-19 pandemic, significantly increases absenteeism compared to 2017, 2018, 2019, 2022 and 2023 (*p* < 0.001). The average difference compared to 2017 is +3.553 days, and compared to 2019 is +3.251 days. The year 2021 continues this trend, with high values of absenteeism significantly higher than in 2017, 2018, 2019, and 2023 (*p* < 0.001) but not significantly different from 2020 (*p* = 0.295), suggesting a continuation of the high level of absenteeism. In the post-pandemic period (2022–2023), absenteeism decreased compared to the pandemic period but remained above pre-pandemic levels. For example, in 2023, sick leave was significantly lower than in 2020 and 2021 (*p* < 0.001) but higher than in 2018 and 2019 (*p* < 0.05) (Table 3).

The data in Table 4 present the average number of days of sick leave by type of respiratory condition, showing that quarantine periods had a higher average of 9168 days. COVID-19 cases had an average of 7806 days of sick leave. Influenza and pneumonia resulted in an average of 5406 days of sick leave, while acute upper respiratory diseases had an average of 3782 days.

As presented in Table 5, workers who received sick leave for COVID-19 have significantly more sick leave days compared to those with acute upper respiratory tract conditions (+4.024 days, *p* < 0.001), other respiratory system diseases (+0.988 days, *p* < 0.001), influenza and pneumonia (+2.400 days, *p* < 0.001). Quarantine causes the most days of absence, even more than COVID-19 (+1.362 days, *p* < 0.001). Those in quarantine had significantly more sick days compared to all other groups. Workers with chronic obstructive pulmonary disease (COPD) did not differ significantly from many other categories in sick leave days, probably due to high variance (high standard errors).

**Figure 2:**
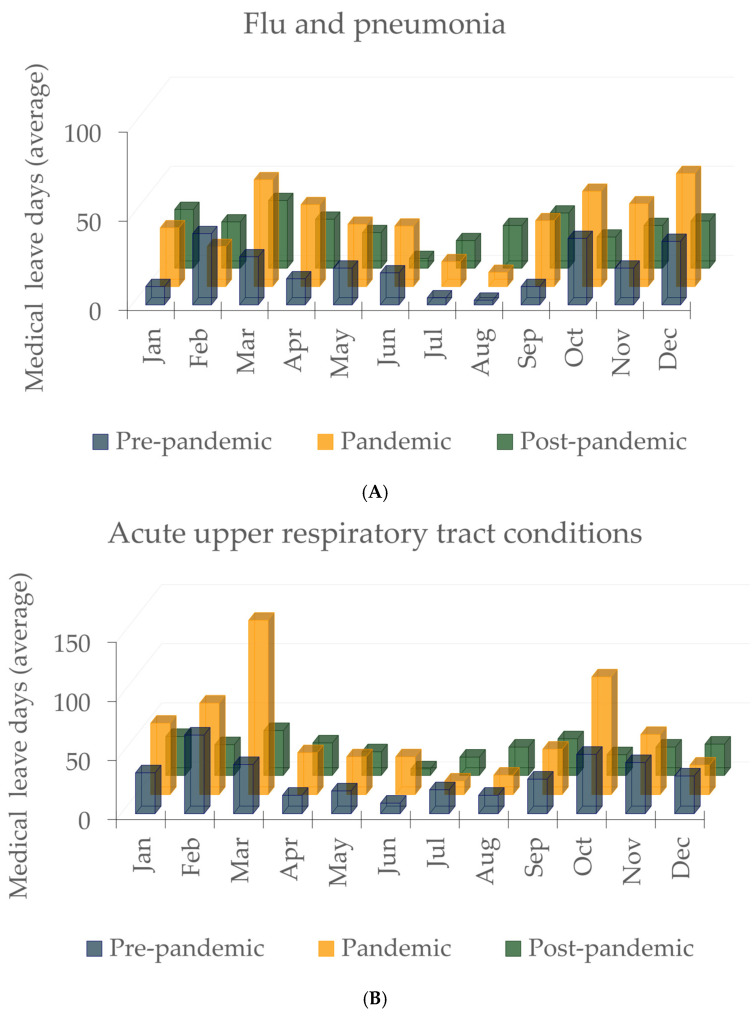
(**A**) Influenza and pneumonia sickness absence evolution before, during, and after the pandemic, 2017–2023. (**B**) Acute upper respiratory tract sickness absence evolution before, during, and after the pandemic, 2017–2023.

Figure 2A shows the evolution of absenteeism due to influenza and pneumonia. In the pre-pandemic period (blue bars), a seasonal peak is observed in December-March, probably due to the flu season. The pandemic period (orange bars) comes with a significant increase in sick days, especially in March, April, and December. The summer months (July and August) had fewer vacation days, probably due to lockdowns and reduced exposure to viruses. In the post-pandemic period (green bars), the distribution of the number of vacation days is somewhat more balanced over the year, with lower values than during the pandemic but higher than before the pandemic.

Acute upper respiratory tract infections (Figure 2B) in the pre-pandemic period (blue bars) follow a seasonal pattern like influenza and pneumonia, with peaks in January, February, and December. The pandemic period (orange bars) expresses peaks in March and October, suggesting that the pandemic had a major impact on upper respiratory infections, probably due to COVID-19 and other circulating viruses. As with influenza and pneumonia, the summer months had fewer vacation days. In the post-pandemic period (green bars), moderate increases start in September, with peaks above pre-pandemic levels.

Table 6 presents data on the sociodemographic and occupational characteristics of respiratory disease-related sick days during 2017–2023. Females consistently accounted for the majority of cases (~85–90%). The 41–50-year-old age group had the highest percentage of sick days, especially in 2019 (73.99%). The <30-year age group had the lowest percentage, often below 10%. Nurses had the highest percentage (~60–75%) of sick leave. Auxiliary staff had the lowest percentage, decreasing to 9.12% in 2023.

The Scheffe post hoc test (Table 7) shows that there is a significant difference between the age group 31–40 and 51–60 (*p* = 0.01).

Scheffe post hoc test (Table 8) shows a significant difference between nurses and physicians in terms of sick leave days (*p* < 0.001).

**Figure 3:**
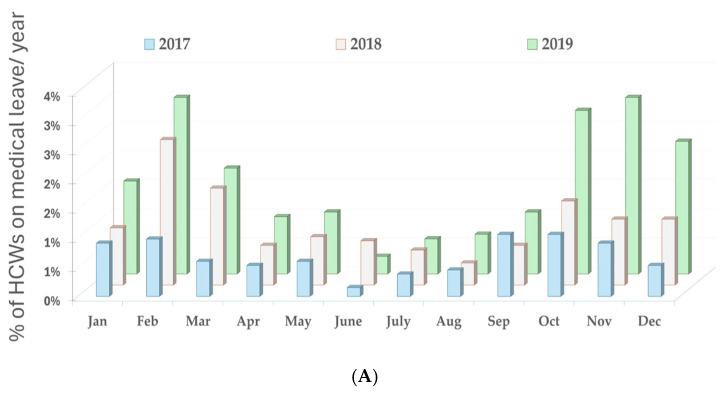

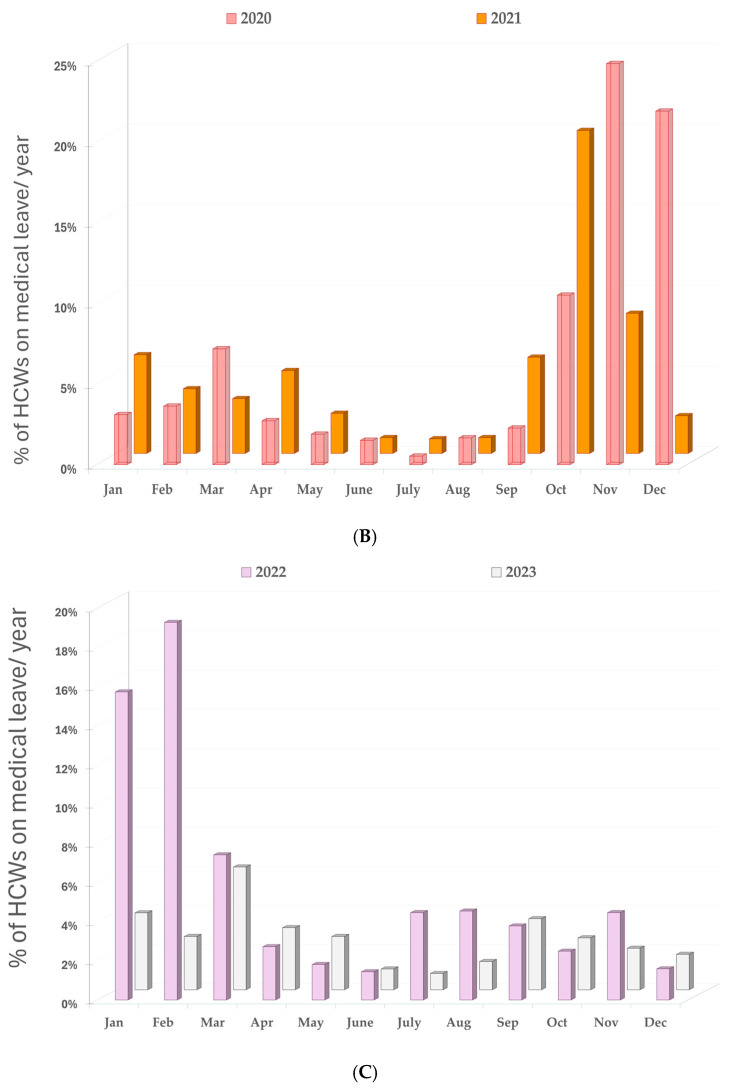
(**A**) Percentage of medical staff on sick leave by month and year before the COVID-19 pandemic. (**B**) Percentage of medical staff on sick leave per month and year during the COVID-19 pandemic. (**C**) Percentage of medical staff on sick leave per month and year after the COVID-19 pandemic.

Analysis of the percentage of medical staff on sick leave by month and year reveals significant changes in absenteeism trends before, during, and after the COVID-19 pandemic. Figure 3A shows low values (<5% of staff) pre-pandemic with peaks in January-March and October-December cold seasons. Increased absenteeism during this period is attributed to flu season and common respiratory viral infections. Figure 3B shows much-increased values during the pandemic (around 20% of staff) with peaks especially in the late October-December period. The year 2020 saw a massive increase in absenteeism, reaching >25% of staff in November and December. In 2021, the values remain high, with a peak in October-November (>20%) but a slight decrease compared to 2020. Post-pandemic (Figure 3C), there are increased values of sick days (around 20% of staff) at the beginning of 2022, after which the percentages of staff on CM tend to decrease to the values of the pre-pandemic period (<5%). The massive increase in sick leave during this period is related to COVID-19 infections, quarantine measures, and the physical and psychological impact on medical staff.

The Generalized Linear Model (GLN) and ANOVA test were used to evaluate the effect that the previously mentioned factors (independent variables) have on sick leave days (dependent variable) (Table 9). The model explains 39.5% of the variance in sick leave days (adjusted R squared = 0.316), and its overall statistical significance is F = 5.002, *p* = 0.001. The dependent variable (sick leave days) and the independent variables—gender, age group, job title, respiratory disorders, and year—show a substantial correlation. With F = 37.511, *p* = 0.001, and a partial η^2^ = 0.063, the respiratory diseases factor has the most significant effect on sick leave days, with 6.3% of the variance being attributed to this variable. Thus, different respiratory diseases clearly affect absenteeism. Confirming that absenteeism trends change over time, especially during and following the COVID-19 epidemic, the year variable is also quite significant (F = 15.796, *p* < 0.001); partial η^2^ = 0.028. Therefore, 2.8% of the variance is attributed to this variable. With “category gender Female” perhaps contributing more to sick leave days depending on the labor structure, the variable gender has a more moderate but significant impact (F = 12.938, *p* = 0.001, partial η^2^ = 0.004). The variable age group (F = 1.420, *p* = 0.225) and variable job title (F = 2.810, *p* = 0.060) do not have a sizeable independent effect. The significant interaction between all variables—Gender × Age Group × Function × Respiratory Disease × Year (F = 1.611, *p* < 0.001, partial η^2^ = 0.168)—highlights the complicated interplay of demographic and occupational factors influencing absenteeism.

**Figure 4:**
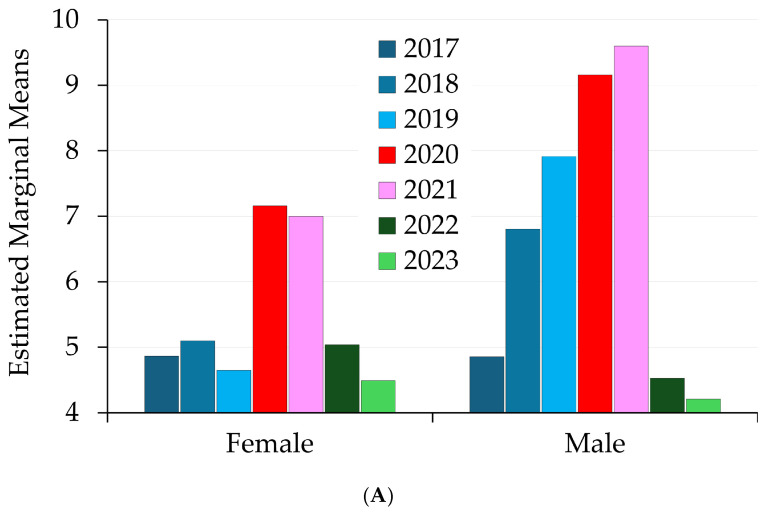

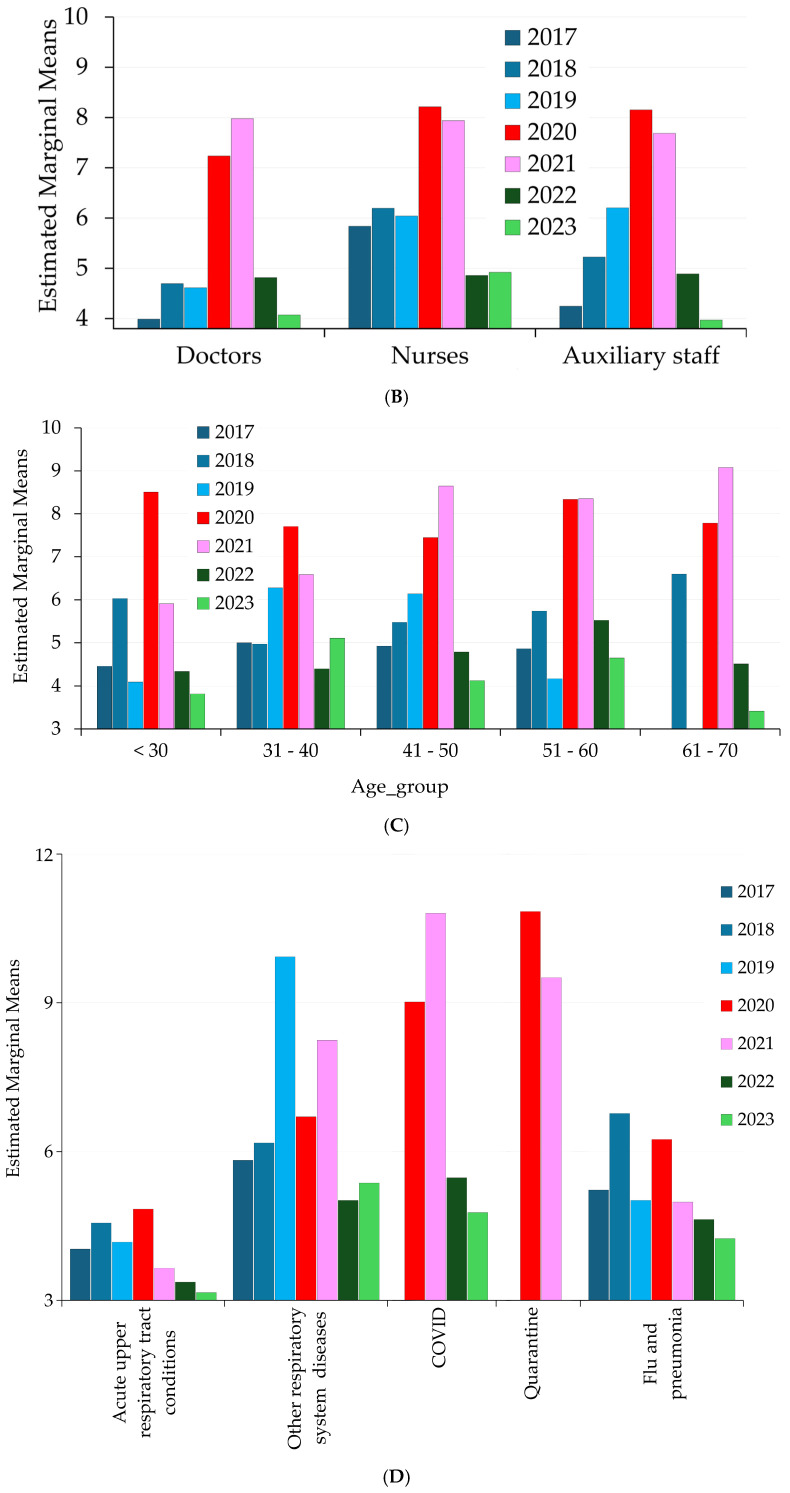
(**A**) The estimated marginal means of sick leave days are based on the independent variable “gender”. (**B**) The estimated marginal means of sick leave days are based on the independent variable “occupation”. (**C**) The estimated marginal means of sick leave days are based on the independent variable “age groups”. (**D**) The estimated marginal means of sick leave days based on respiratory diseases.

The estimated marginal means of sick leave days based on variables such as gender, age group, job title, and disease across years are presented in Figure 4A–D.

Figure 4A shows an increase for both genders in 2020. The decline in sick leave days post-2021 (especially in 2022 and 2023) matches the trend in Table 1.

Figure 4B shows that nurses experienced the most significant increases in sick leave days during the pandemic. A pronounced spike is observed in 2020 and 2021, corresponding to the pandemic.

Figure 4C shows the estimated marginal means of sick leave across different age groups over the years. The age groups below 30 (<30) and 61–70 years old consistently exhibit the lowest sick leave days across most years. Age group 41–50 exhibits the most significant increase in sick leave days during 2020 and 2021, reaching the highest levels among all age groups. For the age group 51–60, sick leave days are lower than for the 41–50 group.

The expected marginal means of medical leave with respiratory disorders over time are shown in Figure 4D. Starting from 2020, there was an abrupt and significant increase in sick leave because of the COVID-19 pandemic. Our data highlight the pandemic’s substantial influence on absences from work and are consistent with its chronology. In 2020, there was an increase in sick days connected to quarantines, which coincided with enforced quarantines during the pandemic’s peak. Possibly due to overlapping COVID-19 symptoms, influenza, and pneumonia, medical leave days exhibit a steady trend with a minor rise in 2020; after the pandemic, the values return to their pre-2020 levels.

## 4. Discussion

Unfortunately, few studies have focused on pediatric HCWs. Concomitant increases in cases of seasonal respiratory diseases (such as influenza) or pandemic COVID-19 are known to have a significant impact on the productivity and safety of pediatric hospital workers [34,35]. It is important to emphasize that the number of sick days due to respiratory illness reflects a relatively stable situation prior to the pandemic (2017–2019). The COVID-19 pandemic had a clear impact during 2020–2021, seen both in the number of sick leave days taken and the types of infections reported (Figure 1). The increase in cases observed in 2020 (Figure 1) is probably connected to the beginning of the COVID-19 pandemic. Even after 2020, respiratory-related sick days remain significant in 2021 and 2022. This tendency indicates that increased absenteeism was driven by direct exposure to infections and the stress associated with high workloads, as reported by nearly all patient care institutions [36]. The decrease in absenteeism in 2023 could be related to both the improved prevention measures and the overall reduction in the impact of COVID-19. The data obtained are in agreement with the published data about the COVID-19 pandemic’s effect on respiratory-related absences among HCWs. During the early stages of the pandemic, before vaccines were widely available, COVID-19 significantly contributed to absenteeism among HCWs [2]. Vaccination has proven to be a critical factor in reducing both the frequency and duration of respiratory-related absences among HCWs [37].

Between 2017 and 2023, the distribution of medical leave days reveals some interesting patterns. The number of days for acute upper respiratory tract infections increased steadily, starting at 213 in 2017 and peaking at 773 in 2020, before fluctuating in subsequent years. The lowest value was in 2017 (213 days). For other respiratory diseases, the trend was similar, with a minimum of 166 days in 2017 and a sharp peak of 927 days in 2020. Influenza and pneumonia followed suit, starting at 130 days in 2017, peaking at 547 in 2020, and tapering off in later years. Chronic obstructive pulmonary diseases showed no recorded leave days from 2017 to 2022 but had 31 days in 2023. Meanwhile, COVID-19 had a massive impact, with no cases before 2020, but increased rapidly to 5021 days of medical leave that year. The sick leave gradually declined to 4590 days in 2021, 3193 days in 2022, and 412 days in 2023. In terms of the total number of medical leave days taken for respiratory infections during this period, the breakdown is as follows: COVID-19, 13,216 days; acute upper respiratory tract infections, 3442 days; other respiratory diseases, 3293 days; quarantine: 2677 days; influenza and pneumonia: 2049 days; and chronic obstructive pulmonary diseases, 31 days. Quarantine-related absences followed a similar trajectory, with no recorded cases before 2020. That year saw 1716 days, which dropped to 961 days in 2021 and then returned to 0 for 2022 and 2023.

In Figure 1, the red segment—representing COVID-19—stands out as the most prominent, highlighting how the pandemic significantly affected absenteeism among medical staff. This finding aligns with Figure 1, which shows a sharp rise in sick leave days in 2020, followed by a slight drop afterward. By 2022–2023, COVID-19 cases had declined substantially, with the reduction in 2023 suggesting that we are adapting to the new public health landscape [38]. As shown in Figure 2, quarantine was an important factor during the pandemic. Even for non-symptomatic subjects, quarantine measures contributed to absenteeism, underscoring the importance of public health protocols [39]. Acute upper respiratory infections (dark blue in Figure 1) and other respiratory diseases (green in Figure 1) remained relatively stable throughout this period, suggesting these illnesses are a consistent cause of sick leave, even outside the pandemic. This finding reflects the consistent vulnerability of healthcare personnel to seasonal respiratory infections [40]. In 2020, there was a noticeable drop in cases of influenza and pneumonia (orange in Figure 1), likely thanks to the strict preventive measures put in place during the pandemic, such as wearing masks and practicing social distancing [37,41]. Published data supported that absenteeism due to respiratory infections could fluctuate due to changes in infection control practices [42]. On the other hand, chronic lung diseases (gray in Figure 1) or other unspecified respiratory conditions (yellow in Figure 1) continued to be a consistent reason for absenteeism [43].

The evolution of the average number of sick days taken for each of the years 2017–2023, shown in Table 1, is consistent with the study hypothesis that absenteeism among pediatric HCWs increased significantly during the pandemic. These results underscore the importance of sustained strategies to protect HCWs, especially during peak periods of respiratory infection.

Using only the year variable (Table 2), we found that 16.1% of the variation in the number of sick days is explained by the year in which they were recorded. The data in Table 3 confirm the strong impact of the pandemic on HCWs and underlines the need for sustained action to protect them. There was a sharp increase in absenteeism in 2020 and 2021, which is significantly different from the pre-pandemic years (2017–2019). We also observed a decreased tendency in absenteeism in the post-pandemic period (2022–2023), which can be explained by the impact of the quarantine and protective measures implemented in hospitals.

Figure 2 shows the absenteeism of HCWs due to respiratory infections as the average number of sick days/HCW on sick leave. It can be seen that the average days in both the pre-pandemic and post-pandemic periods are significantly lower than the average days in the pandemic period. It should also be noted that in the post-pandemic period, the average number of days of sick leave/HCW on sick leave decreases and tends to return to baseline, results that contradict the hypothesis that “absenteeism of pediatric HCWs due to respiratory infections remains above baseline even in the post-pandemic period (2022–2023)”. The steep decline in the post-pandemic period suggests a return to normal, possibly as a result of the recovery of the population’s health. The fact that there are fewer days of sick leave in the post-pandemic period than before the pandemic could indicate either a trend toward avoiding sick leave or an improvement in overall health. These results emphasize the need for proactive strategies to prevent absenteeism among HCWs, especially during the respiratory infection seasons.

From Table 4, it can be seen that there are statistically significant differences between the mean number of days of leave taken for COVID-19 and other respiratory illnesses, including quarantine and influenza (*p* < 0.001). There are also significant differences between days taken for acute respiratory conditions and for influenza and pneumonia. Table 5 shows the significant differences in the number of days lost due to different types of respiratory illness. COVID-19 and quarantine are associated with the longest duration of sick leave. Acute upper respiratory diseases and influenza/pneumonia lead to fewer days of sick leave. These results are key to understanding the impact of each category of illness on sickness absence among HCWs.

It is important to analyze these data in the context of seasonality, as most respiratory infections follow a cyclical pattern, with peaks during the cold seasons of the year. Acute upper respiratory tract infections, influenza, and pneumonia show significant increases in the autumn and winter months, which may explain the observed differences between disease categories. The data also suggest that during the pandemic period, protective measures and quarantine affected the distribution of absenteeism, reducing certain types of seasonal infections but increasing the impact of COVID-19 on sick leave [44,45].

As demonstrated in Figure 2A,B, both influenza and pneumonia, as well as acute upper respiratory tract infections, exhibit a seasonal pattern, with a heightened number of cases observed during the colder months (January-March and October-December). This phenomenon can be attributed to the increased susceptibility to respiratory infections that occur during periods of low temperatures. During the pandemic, there was a significant increase in sick days for both categories, despite the implementation of restrictions, precautionary measures, and an overall increase in respiratory cases [46]. However, following the conclusion of the pandemic, the number of sick days decreased compared to the pandemic period, though it did not fully return to pre-pandemic levels. This trend suggests a lasting impact of the pandemic on respiratory health. While both categories were affected by the pandemic, notable differences emerged in the manifestation of the increase in cases. For instance, influenza and pneumonia exhibited more pronounced peaks in March-April and December, which are traditionally associated with the circulation of influenza viruses. This suggests that, despite preventive measures, influenza and pneumonia continued to affect the population, particularly in the high-incidence months. Acute upper respiratory tract infections, on the other hand, showed extreme increases in March and October. These spikes could be linked to successive waves of the SARS-CoV-2 virus and the spread of other respiratory viruses, which caused an increased need for sick leave [46,47].

The results differ from some published data, which support a significant reduction in cases and related sickness absence during the pandemic due to measures such as wearing masks, social distancing and improved hygiene [21,37,48]. After the pandemic, a gradual return to normal levels of influenza and pneumonia sickness absence was observed. Although levels remain higher than before the pandemic, this may be due to more frequent infections or slower recovery from illness. On the other hand, acute upper respiratory tract infections remain above pre-pandemic levels, data consistent with the literature [40,49]. This trend could indicate changes in the population’s immune response, mutations in circulating viruses, or new sick leave practices [36]. Another possible explanation could be reduced exposure to viruses during the pandemic, leading to increased susceptibility after the pandemic [50]. The impact of the pandemic on sickness absence due to respiratory diseases was significant [51], with large increases for both influenza and pneumonia and acute upper respiratory tract infections. Although the number of sick days decreased after the pandemic, levels did not fully return to previous levels, especially for acute upper respiratory tract infections. This suggests possible changes in the epidemiology of respiratory diseases and the need for continued monitoring of these conditions [52,53,54]. These patterns show how environmental factors play a significant role in the rise in respiratory illnesses, especially during the fall and spring. In order to stay ahead of these peaks, public health initiatives should focus on ramping up efforts, flu vaccination campaigns, and other preventive measures before the critical periods [55,56]. COVID-19-related factors, including vaccine campaigns, mask use, and quarantine regulations, have significantly impacted the prevalence of influenza, pneumonia, and other respiratory illnesses. Published data indicated that adherence to COVID-19 prevention measures (like mask-wearing and hand hygiene) lowers the number of cases of seasonal flu and other respiratory illnesses, including pneumonia and Mycobacterium tuberculosis [57,58]. A study in Germany showed a marked decrease in acute respiratory tract infections (RTIs) and gastrointestinal infections during the pandemic, with influenza diagnoses dropping by 71% in general practices and 90% in pediatric practices [37]. Implementing health protocols such as social distancing and mask-wearing has led to declining influenza and all-cause pneumonia cases. For instance, a study in Taiwan reported a notable decrease in influenza cases following the introduction of these measures [59]. Another study highlighted that the combination of mask-wearing, hand hygiene, and social distancing resulted in a 36% reduction in hospital admissions for chronic obstructive pulmonary disease (COPD) and a 12% decrease for non-COVID-19 pneumonia during the pandemic [43]. Researchers observed COVID-19 co-infections with other respiratory viruses, but the overall trend indicated that preventive measures reduced the severity and incidence of other infections, suggesting a complex interplay between COVID-19 and other respiratory illnesses [60].

As shown in Table 6, female HCWs, especially nurses, have the highest respiratory disease-related medical leave. This finding might reflect the hospital workforce structure or how demanding their roles are. The trends in sick leave days among HCWs, focusing on gender distribution, could differ in other countries, depending on HCWs’ access to paid sick leave [61].

The proportion of sick leave days in the under 30 years old age group (<30) remains under 10%. This result may indicate that younger individuals are less likely to be severely affected by respiratory infections or that their representation in the workforce is smaller than other age groups. The contribution of the age group “31–40 years” to the total number of sick leave days gradually decreased, especially as the age groups 41–50 and 51–60 years took more. This decline may be influenced by stronger immunity or more effective protective measures for this age group. During the pandemic (2020), their proportion decreased to 17.85%, indicating that the older groups (41–50 or 51–60) endured more strain and needed more recovery time. The “41–50 years” age group consistently takes up a significant proportion of sick leave days across all years. This group appears to have endured a higher workload or higher levels of stress, particularly in 2019, when their proportion reached 73.99% of all sick leave days. The 51–60 age group has seen a steady increase in sick leave days since 2019, reaching its highest level in 2023. This finding may suggest increasing health concerns as this group approaches retirement age or a greater susceptibility to respiratory infections in the post-pandemic period. On the other hand, the decrease in the share of sick leave days in the age groups < 30 years and its increase in the age groups 51–60 years could suggest a demographic change in the active medical workforce. The smallest age group represented in the analyzed cohort is the “61–70 years” category, with variable but generally low proportions, indicating a limited presence in the active workforce or more frequent retirement from active duty. The literature data show that HCWs age distribution is influenced by other factors such as job position, the specific health challenges posed by the pandemic, anti-COVID-19 vaccination, and the characteristics of the study’s design [62,63,64].

The results presented in Table 7 emphasize that older HCWs (51–60 years) might be more vulnerable to respiratory infections [65]. Occupational stress and accumulated exposure over time may influence health status [66]. Younger healthcare workers may have faster recovery and fewer sick days [67].

The proportion of sick leave days among physicians slightly increases, from less than 10% (2018) to more than 20% (2023). These data could reflect greater involvement or exposure to patients with respiratory infections. Published data about doctors’ sick leave depend on the context and specific group being examined [50,68]. Nurses consistently have the largest share of sick leave days each year, peaking at 74.61% in 2018 and remaining above 60% in recent years, being the most exposed, involving frequent direct contact with patients [61]. Because of decreased risk exposure or increased adherence to preventive measures, the percentage of sick leave days for auxiliary personnel has decreased over time. The results are confirmed by the Scheffe post hoc test (Table 8). Nurses had more days of sick leave than doctors, which can be explained by the greater direct contact with patients and the greater physical effort and stress in their daily work. Doctors had fewer days of sick leave, possibly because of the pressure to stay at work when ill (presenteeism). Auxiliary staff did not differ significantly from the other categories, which may indicate a different level of exposure to patients.

These data are consistent with the literature. Nurses often have higher physical demands (e.g., lifting patients, long working hours, exposure to infections), which correlate with increased absenteeism due to musculoskeletal problems and fatigue [69]. Healthcare workers, particularly nurses, report higher rates of influenza and respiratory illness than physicians [70]. Nurses often work rotating shifts and night shifts. Research suggests that shift workers have higher rates of sickness absence due to circadian rhythm disruption [71]. In many hospitals, doctors feel cultural pressure to avoid taking sick leave in order to maintain professional credibility [72]. Nurses often experience greater emotional exhaustion due to more frequent exposure to patient distress and less autonomy in decision-making compared to physicians. Burnout among nurses is a well-documented cause of absenteeism among healthcare professionals [73].

The obtained data underlined the need for hospitals to prepare for concurrent outbreaks by aligning infection prevention strategies and resource planning. It may be appropriate to provide specific measures to protect nurses, such as improved access to personal protective equipment, more flexible working hours or additional support during busy periods. Such measures are thought to protect the well-being of both patients and healthcare workers [74]. Developing effective communication routines is essential, and simulations could help improve teamwork efficiency in managing the dual burden of COVID-19 and other respiratory diseases [75,76].

The data presented in Figure 3A–C confirm the hypothesis that the COVID-19 pandemic caused a significant increase in healthcare worker absenteeism, peaking in the autumn and winter of 2020. After the peak in 2020, absenteeism gradually decreased but remained above pre-pandemic levels in 2022–2023, suggesting residual effects on the health of healthcare workers. The impact of COVID-19 on sickness absence was driven by direct infection (COVID-19 confirmed cases), quarantine and isolation measures, physical exhaustion, and burnout associated with high workload. In the pre-pandemic period, absenteeism followed a typical seasonal distribution, with peaks in the cold months. In the pandemic period, this pattern changed, with high peaks even in the spring and summer months, indicating a generalized effect of COVID-19, independent of the usual seasonality of respiratory infections. The seasonality partially returned after the pandemic (2022–2023), but high values in the first months of the year suggest an increased susceptibility of healthcare workers to respiratory infections. The results are consistent with international data showing that healthcare worker absenteeism increased significantly during the pandemic and remained above baseline levels in subsequent years [77,78]. Previous studies confirm that exposure to patients with respiratory infections is high in pediatric hospitals [18,79], which explains the high rate of sickness absence in this sector [61,80].

Further statistical analysis (Table 9 and Figure 4A–D) analyzes the factors affecting the dependent variable “Sick leave days” using a between-subjects design. The model assesses the effects of several factors, such as gender, age group, job title, respiratory diseases, and year, along with their interactions, on sick leave days. These data showed a significant increase in sick leave days in 2020 and 2021, consistent with the COVID-19 pandemic. The combined effect of all independent variables (Table 9) is significant and explains 16.8% of the variance, sustaining the intricacy of interactions among gender, age group, job title, respiratory diseases, and year. The variable “year” accounts for 2.8% (Table 9), a significant contribution as the pandemic generated a major rise in medical sick leave in 2020, which likely caused increased stress, exposure to illness, and absenteeism, particularly among healthcare workers (e.g., nurses and doctors). Respiratory diseases strongly influence sick leave days, explaining 6.3% of the variance. Acute upper respiratory tract diseases, influenza, and pneumonia were associated with higher sick leave days (Figure 4D). Nurses exhibited the highest number of sick leave days, followed by doctors and then auxiliary staff (Figure 4B). Their frontline roles could explain the significant positive impact of sick leave days on the job category nurses [61]. Female HCWs showed consistently higher sick leave days compared to male across all years (Figure 4A). Middle-aged groups (31–40 and 41–50) tend to have more sick leave days than younger (<30) and older (>60) groups (Figure 4C), consistent with literature data [62]. COVID-related sick leave days declined in 2022 and 2023 as the pandemic came under better control. Further, the negative impact on sick leave days of quarantine could reflect the isolation measures.

The particularity of the study comes from the specific setting in which it took place: a regional children’s hospital in the northeastern region of Romania. This hospital treats both emergencies and highly complex cases. As a university hospital, there are medical students, nurses in training, and residents for each specialty. It has inpatient wards and outpatient clinics covering all pediatric care. The pediatric patient, aged between 0 and 18 years, introduces specific variables into the dynamics of medical care. Younger children hospitalized with their parents/guardians expose medical staff to an additional risk of transmission of respiratory infections due to the constant proximity between patients, relatives, and staff. To prevent absenteeism among pediatric HCWs due to respiratory illness, complex interventions are needed, including (i) Vaccination accessible for HCWs—Annual influenza vaccination should be free of charge for pediatric HCWs, with information campaigns on its effectiveness [81]. Although costly, vaccination against pneumococcal and RSV could be promoted for staff constantly exposed to sick children [82]. National campaigns should be supported by the Ministry of Health to increase vaccination coverage [83]. (ii) Effective infection control in hospitals and clinics—Provision of personal protective equipment (PPE)—FFP2 masks, gloves, gowns, all constantly available [84]. Strict hand hygiene by increasing stocks of disinfectant solutions and providing ongoing training on proper hand washing [85]. Frequent disinfection of premises—use of effective cleaning solutions and strict hygiene rules. (iii) Optimizing working conditions—Properly ventilate healthcare premises by using air purification systems where infrastructure allows and encouraging regular room ventilation [86]. Intelligent staff rotation to avoid overburdening during epidemiological peaks [87]. The possibility of working from home for administrative tasks or reducing exposure to patients for staff who can work online (teleworking). Rest and recovery rooms in hospitals for health workers under pressure. Flexibility in planning for sickness—allowing health professionals to stay at home without risk of administrative repercussions. (iv) Screening and early diagnosis of respiratory infections—Free rapid testing for influenza, COVID-19, and other respiratory viruses in health facilities [88]. Monitoring staff health through questionnaires and regular check-ups. Provide easy access to medical consultations for sick health workers so that they do not have to work sick. Respiratory infections in healthcare workers can also be transmitted to patients, leading to nosocomial infections [89,90,91]. Sometimes these respiratory infections can progress to some degree of severity and become superinfected with Klebsiella pneumoniae, a highly pathogenic Gram-negative bacterium [90]. Telemedicine is an effective tool that can help maintain continuity of care. It also helps HCWs manage their health status without needing face-to-face visits when they present symptoms [92,93]. (v) Measures to protect the immunity of HCWs—A diet that is well-balanced (rich in essential nutrients, vitamins, and bioactive compounds [94]) can help HCWs strengthen their immune systems, enhancing their defense against infections and improving overall health. Promote rest breaks and avoid burnout to reduce the risk of immune decline [95]. (vi) Continuous education and awareness-raising—Health workers should be regularly reminded of the importance of immunization, emphasizing the safety and efficacy of available vaccines. Vaccination against respiratory viruses protects HCWs, reduces absenteeism, and increases patient safety [96,97]. Courses and workshops should be held for healthcare professionals on preventing respiratory infections [98], as well as internal campaigns in hospitals and clinics to improve hygiene and compliance with prevention rules. (vii) Collaboration and external support—Psychological support programs for health workers affected by stress and burnout through partnerships with NGOs and exchanging experiences with hospitals in other EU countries [99], in addition to accessing European funding to modernize health infrastructure and implement advanced prevention measures against respiratory diseases. This plan balances the advanced measures used in developed countries with more affordable and feasible solutions in Romania, given the current infrastructure and challenges in the healthcare system.

The study’s limitations: This study provides valuable insights into absenteeism trends among HCWs in a pediatric hospital, but several limitations should be considered. First, the single-center design limits generalizability, as absenteeism patterns may differ across hospitals and regions. Additionally, reliance on administrative records may lead to underreporting or misclassification, while presenteeism (HCWs working while sick) was not assessed, potentially underestimating the true burden of illness. Future research should incorporate self-reported surveys to address this gap. Several confounding factors were not controlled, including vaccination status, comorbidities, workload intensity, and changes in sick leave policies, all of which can influence absenteeism trends. Moreover, inconsistencies in sick leave reporting across hospital departments may affect comparisons. Standardized data collection would improve accuracy. The study does not differentiate between short-term and long-term absences or track recurrent sick leave, limiting insights into chronic absenteeism. Finally, selection bias and residual confounding may still be present despite multivariate analysis. Refining statistical methods to handle missing data, seasonality, and outliers would enhance reproducibility. Despite these limitations, this study provides important insights. Future research should integrate multi-center data and key covariates to improve accuracy and applicability.

## 5. Conclusions

With post-pandemic levels still higher than before, the data highlight how respiratory illnesses and the COVID-19 outbreak have significantly increased absenteeism among healthcare workers. Conducted at a regional pediatric hospital in Romania, the study highlights the need for specially designed protocols to protect healthcare workers on the one hand and pediatric patients and their families on the other. The convergence of rising COVID-19 and respiratory illness cases can affect pediatric hospital resources and compromise standard functionality. Thus, preventive approaches, including immunization, HCW health monitoring, and education, are vital to lower absenteeism, preserve a strong workforce, and guarantee ongoing, high-quality treatment during epidemics.

## Figures and Tables

**Table 1:** Comparison of absenteeism by year.

Estimates				
Dependent Variable: Sick Leave Days				
Year	Mean	Std. Error	95% Confidence Interval	
			Lower Bound	Upper Bound
2017	4.769	0.371	4.040	5.497
2018	5.361	0.297	4.779	5.943
2019	5.071	0.242	4.596	5.546
2020	8.322	0.117	8.092	8.552
2021	8.134	0.136	7.867	8.400
2022	5.028	0.127	4.779	5.277
2023	4.332	0.187	3.966	4.698

**Table 2:** Effect of the “year” variable on absenteeism.

Univariate Tests						
Dependent Variable: Sick Leave Days						
	Sum of Squares	df	Mean Square	F	Sig.	Partial Eta Squared
Contrast	10,799.625	6	1799.938	120.760	<0.001	0.161
Error	56,102.713	3764	14.905			

The F test evaluates the effect of YEAR. It is based on the linearly independent pairwise comparisons among the estimated marginal means.

**Table 3:** Years’ pairwise comparisons.

Years’ Pairwise Comparisons						
Dependent Variable: Sick Leave Days						
(I) Year	(J) Year L	Mean Difference (I-J)	Std. Error	Sig. ^b^	95% Confidence Interval for Difference ^b^	
					Lower Bound	Upper Bound
2020	2017	3.553 *	0.390	<0.001	2.789	4.317
	2018	2.961 *	0.319	<0.001	2.335	3.587
	2019	3.251 *	0.269	<0.001	2.723	3.779
	2021	0.188	0.180	0.295	−0.164	0.540
	2022	3.293 *	0.173	<0.001	2.954	3.633
	2023	3.990 *	0.220	<0.001	3.558	4.422
2021	2017	3.365 *	0.396	<0.001	2.590	4.141
	2018	2.773 *	0.327	<0.001	2.132	3.413
	2019	3.063 *	0.278	<0.001	2.518	3.607
	2020	−0.188	0.180	0.295	−0.540	0.164
	2022	3.105 *	0.186	<0.001	2.741	3.470
	2023	3.802 *	0.231	<0.001	3.349	4.254
2022	2017	0.260	0.393	0.508	−0.510	1.030
	2018	−0.333	0.323	0.303	−0.966	0.301
	2019	−0.043	0.274	0.876	−0.579	0.494
	2020	−3.293 *	0.173	<0.001	−3.633	−2.954
	2021	−3.105 *	0.186	<0.001	−3.470	−2.741
	2023	0.696 *	0.226	0.002	0.254	1.139
2023	2017	−0.437	0.416	0.294	−1.252	0.378
	2018	−1.029 *	0.351	0.003	−1.717	−0.342
	2019	−0.739 *	0.306	0.016	−1.339	−0.140
	2020	−3.990 *	0.220	<0.001	−4.422	−3.558
	2021	−3.802 *	0.231	<0.001	−4.254	−3.349
	2022	−0.696 *	0.226	0.002	−1.139	−0.254

Based on estimated marginal means. *: The mean difference is significant at the 0.05 level. ^b^: Adjustment for multiple comparisons: Least Significant Difference (equivalent to no adjustments).

**Table 4:** Average number of sick days by type of respiratory disease.

Estimates				
Dependent Variable: Sick Leave Days				
Category_Respiratory Diseases	Mean	Std. Error	95% Confidence Interval	
			Lower Bound	Upper Bound
Acute upper respiratory tract conditions	3.782	0.126	3.535	4.030
Other unspecified conditions	7.444	1.271	4.953	9.936
Other respiratory system diseases	6.818	0.173	6.478	7.158
COVID	7.806	0.093	7.625	7.988
Chronic obstructive pulmonary diseases	6.200	1.705	2.857	9.543
Quarantine	9.168	0.223	8.730	9.605
Flu and pneumonia	5.406	0.196	5.022	5.790

**Table 5:** Paired comparisons of sick leave days between different categories of respiratory diseases.

Pairwise Comparisons						
Dependent Variable: Sick Leave Days						
Scheffe						
(I) Category_Respiratory Diseases	(J) Category_Respiratory Diseases	Mean Difference (I-J)	Std. Error	Sig. ^b^	95% Confidence Interval for Difference ^b^	
					Lower Bound	Upper Bound
Acute upper respiratory tract conditions	Other unspecified conditions	−3.662 *	1.277	0.004	−6.166	−1.158
	Other respiratory system diseases	−3.035 *	0.215	<0.001	−3.456	−2.615
	COVID	−4.024 *	0.157	<0.001	−4.331	−3.717
	Chronic obstructive pulmonary diseases	−2.418	1.710	0.157	−5.770	0.935
	Quarantine	−5.385 *	0.256	<0.001	−5.888	−4.883
	Flu and pneumonia	−1.624 *	0.233	<0.001	−2.081	−1.167
COVID	Acute upper respiratory tract conditions	4.024 *	0.157	<0.001	3.717	4.331
	Other unspecified conditions	0.362	1.274	0.776	−2.137	2.860
	Other respiratory system diseases	0.988 *	0.197	<0.001	0.603	1.374
	Chronic obstructive pulmonary diseases	1.606	1.708	0.347	−1.742	4.954
	Quarantine	−1.362 *	0.242	<0.001	−1.835	−0.888
	Flu and pneumonia	2.400 *	0.217	<0.001	1.975	2.825

Based on estimated marginal means. *: The mean difference is significant at the 0.05 level. ^b^: Adjustment for multiple comparisons: Least Significant Difference (equivalent to no adjustments).

**Table 6:** Sociodemographic and occupational characteristics of respiratory diseases—related sick leave days in the period 2017–2023.

Year	2017	2018	2019	2020	2021	2022	2023
Number of Medical Leave Days	515	906	1288	9004	6572	4636	1854
Gender							
Female (%)	84.66	87.53	89.67	84.91	89.49	85.01	86.84
Male (%)	15.34	12.47	10.33	15.09	10.51	14.99	13.16
Age group							
<30 (%)	9.51	6.51	2.56	5.56	3.32	7.61	3.61
31–40 (%)	30.68	18.65	22.13	17.85	14.06	14.32	17.37
41–50 (%)	36.50	48.12	73.99	46.06	46.93	45.58	37.00
51–60 (%)	23.30	19.98	1.32	28.20	31.88	30.91	39.10
61–70 (%)	0.00	6.73	0.00	2.33	3.82	1.57	2.91
Occupation							
Doctors (%)	25.44	8.28	11.88	18.05	18.84	22.76	22.76
Nurses (%)	57.28	74.61	71.20	70.90	69.55	60.40	68.12
Auxiliary staff (%)	17.28	17.11	16.93	11.05	11.61	16.85	9.12

**Table 7:** The differences in absenteeism by age group.

Multiple Comparisons						
Dependent Variable: Sick Leave Days						
Scheffe						
(I) Category_Age	(J) Category_Age	Mean Difference (I-J)	Std. Error	Sig.	95% Confidence Interval	
					Lower Bound	Upper Bound
<30	31–40	0.03	0.335	1.000	−1.00	1.07
	41–50	−0.32	0.310	0.897	−1.28	0.63
	51–60	−0.73	0.321	0.271	−1.72	0.26
	61–70	−0.48	0.518	0.932	−2.07	1.12
31–40	<30	−0.03	0.335	1.000	−1.07	1.00
	41–50	−0.35	0.191	0.486	−0.94	0.23
	51–60	−0.076 *	0.209	0.010	−1.41	−0.12
	61–70	−0.51	0.457	0.871	−1.92	0.90
41–50	<30	0.32	0.310	0.897	−0.63	1.28
	31–40	0.35	0.191	0.486	−0.23	0.94
	51–60	−0.41	0.165	0.191	−0.92	0.10
	61–70	−0.16	0.439	0.998	−1.51	1.20
51–60	<30	0.73	0.321	0.271	−0.26	1.72
	31–40	0.76 *	0.209	0.010	0.12	1.41
	41–50	0.41	0.165	0.191	−0.10	0.92
	61–70	0.25	0.447	0.989	−1.12	1.63
61–70	<30	0.48	0.518	0.932	−1.12	2.07
	31–40	0.51	0.457	0.871	−0.90	1.92
	41–50	0.16	0.439	0.998	−1.20	1.51
	51–60	−0.25	0.447	0.989	−1.63	1.12

Based on observed means. The error term is Mean Square (Error) = 17.692. *: The mean difference is significant at the 0.05 level.

**Table 8:** Differences in absenteeism between different occupations.

Multiple Comparisons						
Dependent Variable: Sick Leave Days						
Scheffe						
(I) Category_Occupation	(J) Category_Occupation	Mean Difference (I-J)	Std. Error	Sig.	95% Confidence Interval	
					Lower Bound	Upper Bound
Doctor	Nurse	−0.76 *	0.172	<0.001	−1.18	−0.33
	Auxiliar staff	−0.46	0.242	0.164	−1.05	0.13
Nurse	Doctor	0.76 *	0.172	<0.001	0.33	1.18
	Auxiliar staff	0.30	0.208	0.363	−0.21	0.80
Auxiliar staff	Doctor	0.46	0.242	0.164	−0.13	1.05
	Nurse	−0.30	0.208	0.363	−0.80	0.21

Based on observed means. The error term is Mean Square (Error) = 17.664. *: The mean difference is significant at the 0.05 level.

**Table 9:** Tests of between-subjects effects.

Source of Variation	Type III Sum of Squares	df	Mean Square	F-Value	Sig. (*p*-Value)	Partial Eta Squared (Effect Size)
**Model (Overall Effect) or Corrected Model**	26,457.06	436	60.681	5.002	0.001	**0.395**
**Intercept (Baseline Effect)**	6718.29	1	6718.29	553.804	0.001	0.142
**Gender**	156.951	1	156.951	12.938	0.001	0.004
**Age Group**	68.898	4	17.224	1.42	0.225	0.002
**Job Title**	68.176	2	34.088	2.81	0.06	0.002
**Respiratory Diseases**	2730.32	6	455.053	37.511	0.001	0.063
**Year (Time Effect)**	1149.71	6	191.618	15.796	0.001	0.028
**Interaction: Gender × Age × Job Title × Diseases × Year**	8151.72	417	19.548	1.611	0.001	0.168
**Error (Unexplained Variation)**	40,445.28	3334	12.131	-	-	-
**Total**	229,671	3771	-	-	-	-
**Corrected Total**	66,902.34	3770	-	-	-	-

## Data Availability

All data are available from the first author and the corresponding author.

## Abbreviation

The following abbreviation is used in this manuscript:

HCWs	Health care workers
